# Age-Related Increase in the Number of Oligodendrocytes Is Dysregulated in Schizophrenia and Mood Disorders

**DOI:** 10.1155/2011/174689

**Published:** 2011-07-03

**Authors:** Victor Vostrikov, Natalya Uranova

**Affiliations:** Laboratory of Clinical Neuropathology, Mental Health Research Center, Moscow 115522, Russia

## Abstract

The postnatal maturation of the human prefrontal cortex is associated with substantial increase of number of oligodendrocytes. Previously, we reported decreased numerical density of oligodendrocytes in the prefrontal cortex in schizophrenia and mood disorders. To gain further understanding of the role oligodendrocytes in pathogenesis of schizophrenia and mood disorders, we examined the effect of the age on the number of oligodendrocytes in the prefrontal cortex in schizophrenia, bipolar disorder, and major depressive disorder. We revealed the age-related increase in numerical density of oligodendrocytes in layer VI and adjacent white matter of BA10 and BA 9 in normal controls but not in schizophrenia, bipolar disorder, and major depressive disorder. The absence of normal increase in the number of oligodendrocytes in gray and white matter with age in schizophrenia and mood disorders suggests that age-related process of oligodendrocyte increase is dysregulated in schizophrenia and mood disorders.

## 1. Introduction

Disconnection among different brain regions is believed to contribute to the abnormal functioning of neural networks and has been postulated to be central to the pathophysiology of schizophrenia [1–3]. The neurobiological substrate of these connectivity abnormalities remains unknown, but recent evidence suggests that abnormalities in myelination and altered oligodendrocyte number and function may be prominent contributors to schizophrenia. Indirect evidence for disturbed structural connectivity in schizophrenia has been obtained from functional neuroimaging and electrophysiologic studies. Imaging and postmortem studies provide converging evidence that patients with schizophrenia have a dysregulated process of frontal lobe myelination.

With growing evidence of disturbed connectivity in schizophrenia, myelin pathology has been a target of recent postmortem studies. As oligodendrocytes are essential for the production of myelin, disturbed myelination might be caused by dysfunctional oligodendrocytes or a reduced number of oligodendroglial cells. Many postmortem studies are focusing on the investigation of numerical density of oligodendroglial cells and structural alterations of myelin sheets. Evidence for the involvement of oligodendrocytes and myelin in the pathophysiology of schizophrenia has come from analysis of postmortem tissue subjects with schizophrenia using protein [4, 5] and gene expression studies [5–11], light, and electron microscopic studies [12–14], and in vivo neuroimaging studies [15–19].

The first direct evidence of oligodendrocyte deficit in schizophrenia was obtained from a series of studies performed in our laboratory [12, 13, 20].

We used the Stanley brain collection to estimate Nv of oligodendrocytes in the prefrontal cortex (BA9) in schizophrenia, bipolar disorder and major depression and our own Mental Health Research Center brain collection to estimate numerical density (Nv) of oligodendrocytes in the prefrontal cortex (BA10) in schizophrenia. Reduced Nv of oligodendrocytes was detected in BA9 in layer VI in schizophrenia, bipolar disorder and major depression [13] and in layer VI of BA10 and in adjacent white matter in schizophrenia [21]. Similarly, conducting a stereologic analysis of numbers, densities, and spatial distribution of oligodendrocytes, Hof et al. [22] also found a decrease in total number in cortical layer III and white matter in the superior frontal gyrus in schizophrenics compared to control cases.

In another morphometric study, we demonstrated decreased number of pericapillary oligodendrocytes in layer V BA10 of prefrontal cortex in schizophrenia [23]. We also found that the number of pericapillary oligodendrocytes per unit capillary length positively and significantly correlated with age in control group but not in schizophrenic group [23].

There are only few data on the effect of age on Nv of oligodendrocytes in normal controls and mental illnesses. Byne et al. [24] using the Stanley brain collection demonstrated a significant deficit of oligodendrocytes in thalamic nuclei in schizophrenia and in bipolar disorder. Both psychiatric groups compared to normal controls exhibited an attenuation of an age-related increase in the number of oligodendrocytes.

There is an apparent temporal relationship between myelination and the onset of schizophrenia. Myelination in the frontal and temporal lobes, brain regions consistently implicated in the pathophysiology of schizophrenia, occurs during late adolescence to early adulthood corresponding closely to the peak incidence of schizophrenia onset [25–30].

We hypothesized that the effect of age on Nv of oligodendrocytes might be different in controls and psychiatric groups. The aim of the study was to reveal the effect of age on numerical density of oligodendrocytes in the prefrontal cortex in two brain collections—Stanley Foundation Neuropathology Consortium (SFNC) and Mental Health Research Center (MHRC) collections to learn more about the origin of previously found deficit of oligodendroglial cells in the prefrontal cortex in major psychiatric disorders.

## 2. Methods

The SFNC consisted of 60 subjects: 15 schizophrenia, 15 bipolar disorder, 15 major depression, and 15 unaffected controls. Clinical records were obtained and DSM-IV diagnoses were made by psychiatrists from the SFNC. Routine neuropathological examinations were also conducted on each case by neuropathologists from the SFNC. Demographic data are given in Table 1.

The MHRC brain collection consisted of 64 subjects: 32 schizophrenia and 32 normal controls subjects matched by age, gender, and postmortem delay. Brains for this collection were obtained within short postmortem delay (mean 6 h). Ethical considerations in obtaining and using human autopsy material were informed by the Ethics Committee of the MHRC, the Russian Academy of Medical Sciences. Consent for autopsy and research was taken from family members. Demographic data are given in Table 2. Clinical records were obtained, and ICD-10 diagnoses were made by psychiatrists from MHRC. Causes of death in the schizophrenia group were the same as those in controls: coronary vascular disease, pulmonary embolism, myocardial infarction, pneumonia, and cardiac failure. Brains were excluded from the study if there was evidence of neurological damage, stroke, substantial drug, or alcohol abuse, or if there were changes characteristic of Alzheimer's disease or Parkinson's disease. Only the left hemispheres of brains were taken for study.

For estimation Nv of oligodendrocytes in BA9 and BA10 paraffin sections of 10 *μ*m thickness were obtained from each case using systematic random sampling [31]. Sections were Nissl stained according to the standard method. BA9 and BA10 were verified by us using the morphological criteria established by Rajkowska and Goldman-Rakic [32]. Layer VI was identified by its location between layer V, which contains large pyramidal neurons, and adjacent white matter. Oligodendroglial cells were identified as profiles with a small round nucleus, with densely and homogenously staining and a narrow rim of cytoplasm [33]. In a similar study [22], close agreement was found between such profiles and immunocytochemically identified oligodendrocytes. Ten sections from each brain were used for estimation of the Nv of oligodendrocytes in layer VI and adjacent white matter. Nv of oligodendrogliocytes was estimated by optical dissector method [34]. Sections were viewed using a Carl Zeiss Axio Imager M1 light microscope with 100 × (N.A. = 1.40) oil immersion objective lens, which was connected via AxioCam MRc5 digital camera to a video monitor. Optical sectioning by AxioVision Module *Z*-stack was used for creation series *Z*-stack images. An unbiased sampling frame (frame size 0.05 × 0.065 mm = 0.00325 mm^2^)was superimposed on the images of layer VI and of adjacent white matter with random start, 10 fields with equal interval (three frames) between fields were counted from each section, and 100 fields per each case were counted. One hundred dissectors were counted per layer VI and 100 dissectors per white matter per case. The thickness of the counting brick was 0.75 *μ*m, and the total depth of the optical dissector was 6.75 *μ*m with guards above and bellow *∼*0.5 *μ*m.

Numerical density (Nv) of oligodendroglial cells was estimated using the formula: Nv*** = **Q/v *(dis), where *Q* is the average number of cell nuclei counted per dissector and *v*(dis) is the volume of the disector: *v*(dis) = *a*[frame] × *h*, where *“a”* is area of frame and *“h”* is dissector height. Nv of oligodendroglial cells was estimated as number of oligodendroglial cells in 0.001 mm^3^. Values for coefficient of error of the oligodendroglial density estimates in layer VI and adjacent white matter were consistently low, with group mean scores of <2.5% for layer VI and <1% for white matter.

For estimation of number of pericapillary oligodendrocytes in BA10 (layer V) the 30 paraffin sections of 20 mm thickness were prepared from each case. Ten sections from the series were sampled in systematic random manner for analysis. These sections were stained with Luxol-fast blue and cresyl violet for visualization of capillaries and of pericapillary oligodendrocytes. BA10 was verified, and layer V was identified as containing large pyramidal neurons. A Carl Zeiss Axio Image M1 light microscope with a 25/0.45 objective, which was connected via AxioCam MRc5 digital camera to a video monitor, was used. Ten rectilinear capillary segments from layer V were systematically random sampled from each section. In this manner, 100 rectilinear capillary segments were sampled per brain, and a total of 3200 capillary segments per group were sampled. The length of each segment was measured, the number of oligodendrocytes visible alongside each segment was counted, and oligodendrocyte densities were expressed as the number of oligodendrocytes per 0.01 mm of capillary length.

Statistical analysis was performed using STATISTICA 6 software package for Windows (StatSoft. Inc, Tusla, Okla, USA). Comparisons of number of oligodendrocytes to age in schizophrenia and normal controls were performed using multiple linear regression analysis. Statistical group comparisons between patients and the control groups were made using one-way analysis of variance (ANOVA).

## 3. Results and Discussion

**Figure 1:**
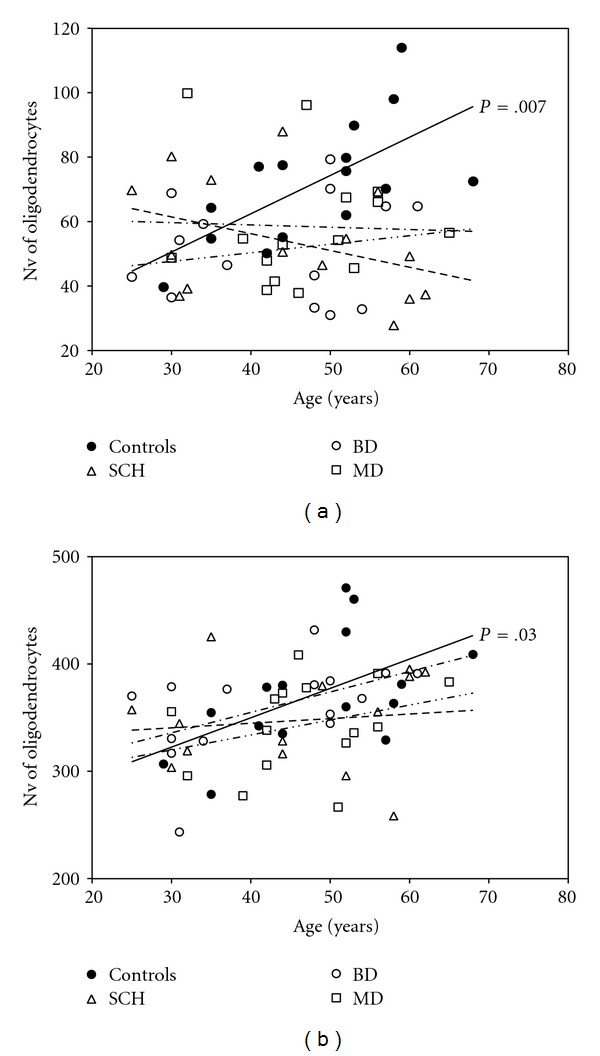
Plots summarizing results of regression analysis in BA9. There is a significant positive correlation between Nv of oligodendrocytes and age in controls in layer VI (*P* = .007) (a) and in adjacent white matter (*P* = .03) (b) but no significant correlations in schizophrenia, bipolar disorder, and major depression.

Regression analyses examining the relationship between age and Nv of oligodendrocytes showed in controls a significant diagnosis by age interaction in layer VI of BA9 (*F* = 10.05, *df* = 1.13, *P* = .007) and in adjacent white matter (*F* = 5.48, *df* = 1.13, *P* = .03). In contrast, none of age-related changes were showed in schizophrenia, bipolar disorder, and major depression (all *P* > .05) (Table 3) (Figure 1).

Regression analyses of the relationship between neuroleptics dosage and Nv of oligodendrocytes in schizophrenic cases showed no significant neuroleptics by age interaction in layer VI of BA9 (*F* = 0.6, *df* = 1.12, *P* = .5) and in adjacent white matter (*F* = 0.15, *df* = 1.12, *P* = .7). Also, there was no significant neuroleptics by age interaction in bipolar disorder in layer VI of BA9 (*F* = 0.01, *df* = 1.10, *P* = .9) and in adjacent white matter (*F* = 3.44, *df* = 1.10, *P* = .09).

**Figure 2:**
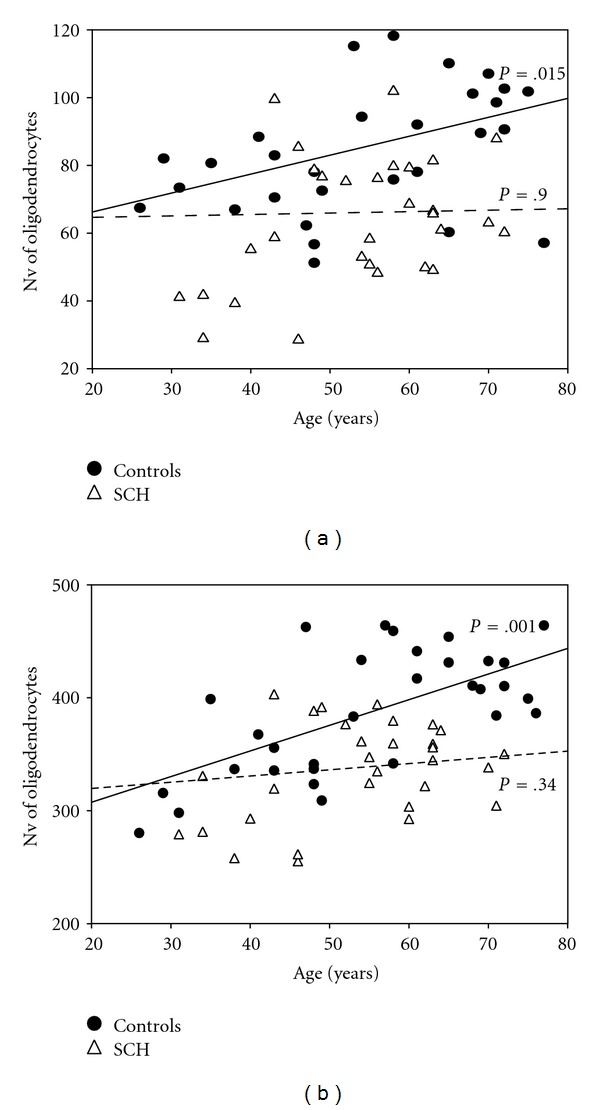
Plots summarizing results of regression analysis in BA10. There is a significant positive correlation between Nv of oligodendrocytes and age in controls in layer VI (*P* = .015) (a) and adjacent white matter (*P* = .001) (b) but no significant correlations in schizophrenia.

Regression analyses examining the relationship between age and Nv of oligodendrocytes showed in controls a significant diagnosis by age interaction in layer VI of BA10 (*F* = 6.55, *df* = 1.30, *P* = .015) and adjacent white matter (*F* = 20.44, *df* = 1.30, *P* < .001). In contrast, none of meaningful age-related change showed in schizophrenia (*F* = 0.02, *P* = .9, *F* = 0.90, *P* = .34, resp.) (Table 4) (Figure 2).

Regression analyses examining the relationship between neuroleptics dosage and Nv of oligodendrocytes in schizophrenia showed no significant neuroleptics by age interaction in layer VI of BA10 (*F* = 3.15, *df* = 1.30, *P* = .09) and in adjacent white matter (*F* = 0.18, *df* = 1.30, *P* = .7).

**Figure 3:**
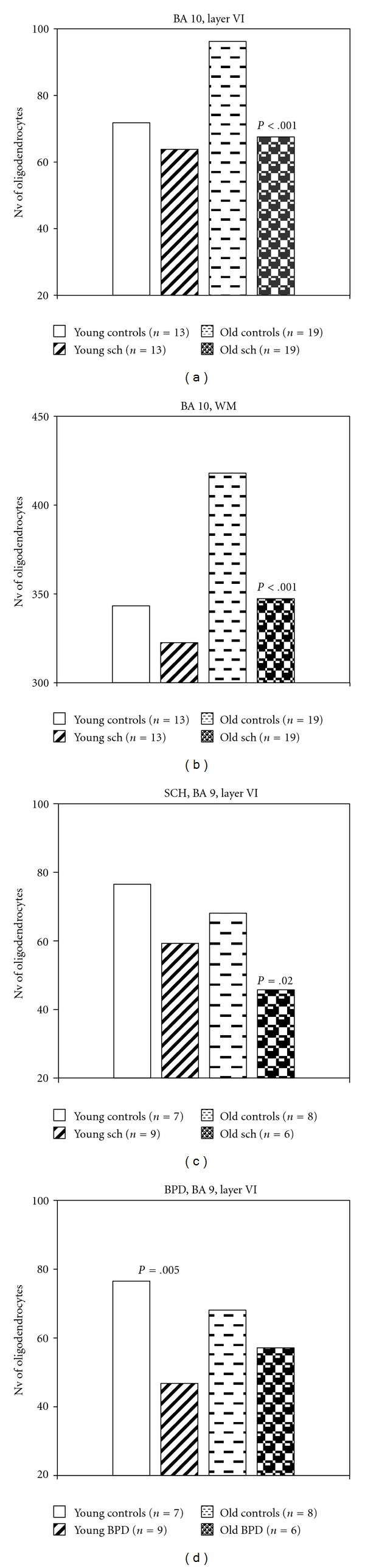
Plots summarizing results of comparisons of young and elderly patient subgroups with young and elderly controls.

Comparisons of young (<50 years) and elderly patient subgroups (>50 years) with young and elderly controls showed that only elderly patients with schizophrenia had significantly lower oligodendrocyte number as compared to elderly controls (in BA10 layer VI (*F* = 26.78, *df* = 1.36, *P* < .001)), in adjacent white matter (*F* = 51.24, *df* = 1.36, *P* < .001), in BA9 layer VI (*F* = 7.28, *df* = 1.12, *P* = .02) (Figures 3(a), 3(b) and 3(c)).

Significant reduction of oligodendrocyte density only was found in young patients with bipolar disorder as compared to young controls (*F* = 11.30, *df* = 1.14, *P* = .005) (Figure 3(d)).

Results of our study revealed a significant age-related increase in Nv of oligodendrocytes in layer VI and in white matter both in BA9 and BA10 in control groups. It is of interest to note that previously the age-related increase number of pericapillary oligodendrocytes in layer V in BA10 was also revealed in controls [23]. Of note, such an increase was not observed in age-matched psychiatric groups.

Our results obtained in normal control groups are consistent with the results of imaging studies on myelination of normal subjects. Bartzokis et al. [19] reported that the normal age-related development of the frontal and temporal lobes in adulthood is dysregulated in adults with schizophrenia primary due to a lack of normal myelination. van Haren et al. [35] proposed that cerebral gray matter volume loss in patients with schizophrenia was characterized by the absence of the normal curved trajectory of volume change with age that was present in healthy subjects. These data together with the results of postmortem studies suggest that age-related increase in oligodendrocyte number and progressive myelination that are evidenced in normal controls may be disrupted in psychiatric patients. A significant age-associated reduction in fractional anisotropy, progressive decrement of white matter volume in frontal lobe, and the relationships between white matter alterations and severity of symptoms were found in patients with schizophrenia in contrast to controls [36–39]. Our results are also in agreement with the data obtained in the monkey neocortex [40] where a substantial increase in the numbers of oligodendrocytes was found over the monkey's life span. An increase in the number of oligodendrocyte with aging has been also reported in the optic nerve, in the visual cortex [41, 42]. Severity of age-related myelin alterations in visual cortex correlated significantly with cognitive impairment index due to reduced conduction velocity along the affected nerve fibers [42, 43].

The increase of oligodendrocytes number and extensive myelination of the human brain makes myelin maintenance and repair especially critical for supporting our high cognitive processing speed [19, 44]. Importantly, that reduction of fractional anisotropy in different white matter tracts was associated with cognitive deficits [45–49]. Thus, these data together with our results suggest that reduced oligodendrocyte density might be associated with cognitive dysfunction in schizophrenia and in bipolar disorder.

In the present study, only elderly patients with schizophrenia had significantly lower oligodendrocyte number as compared to elder controls. Elderly schizophrenia patients frequently develop more severe cognitive disturbances that younger ones. In these subjects reported reduced fractional anisotropy in frontotemporal clusters associated with frontal gray matter reduction and “frontal” cognitive deficits [50], though Frisoni et al. [51] found that orbitofrontal/cingulate region had low gray matter volume in elderly schizophrenia patients not associated with cognitive abnormalities. Neuroimaging studies demonstrated lower prefrontal gray matter volume in schizophrenia in chronic but not in first episode schizophrenia patients [52]. Patients with chronic schizophrenia showed widespread cortical thinning that particularly affected the prefrontal cortex [53–56], decreased fractional anisotropy in thalamofrontal white matter [57], lower levels of glutamate/glutamine and N-acetylaspartate compared to healthy controls and first-episode patients [58], abnormally expressed NMDA receptor subunits in elderly schizophrenia patients [59], and hypoperfusion of the prefrontal cortex [60].

On the contrary, gray and white matter volumes of prefrontal cortex were significantly smaller only in young adulthood bipolar disorder patients relative to healthy control subjects [61–64]. Decreased N-acetylaspartate was detected in the dorsolateral prefrontal cortex of young bipolar patients [65]. These findings suggest that prefrontal white matter abnormalities are present early in bipolar disorder and may consist largely of axonal disorganization, and white matter pathology may represent an early marker of bipolar disorder [66].

Our results that significant reduction of oligodendrocyte density was found in young patients with bipolar disorder as compared to young controls are in agreement with these in vivo data. They are also in line with the data obtained from other postmortem studies reported that patients with bipolar disorder demonstrated decreased neuronal size in the orbito-frontal cortex [67] and reductions in oligodendrocyte density in the prefrontal cortex [13, 14] and the number, size, and density of glial cells [68–70].

Maturity of oligodendrocytes is influenced by genetic and epigenetic factors. DNA microarray analysis repeatedly demonstrated downregulation of oligodendrocyte- and myelin-related genes [6, 8]. Most of these data were obtained on Mount Sinai and Stanley collections containing mostly elderly subjects. On the other side, Mitkus et al. [71] using brain collection containing younger cases of schizophrenia and controls did not confirmed downregulation of oligodendrocyte-related genes, but they found that individuals carrying risk-associated alleles in oligodendrocyte-related genes had relatively lower transcript levels. DTI studies of subjects at increased risk for bipolar disorder showed reduced fractional anisotropy in the superior frontal tracts [72] and decreased gray matter density. Bipolar disorder and schizophrenia share common chromosomal susceptibility loci and many risk-promoting genes. Thus, these data illustrate the importance of genetic factors in oligodendrocyte abnormalities in schizophrenia and in bipolar disorder.

Recently, epigenetic mechanisms facilitating oligodendrocyte development, maturation, and aging have been reported [73]. It is of interest to note that Iwamoto et al. [10] in BA10 of the prefrontal cortex of the Stanley brain collection detected reduced expression of SOX 10, an oligodendrocyte-specific transcription factor, and correlation between DNA methylation status of SOX 10 with its downregulation and oligodendrocyte dysfunction in schizophrenia. It is known that oligodendrocytes are very sensitive to oxidative stress [69, 74, 75] and glutamate toxicity [69, 76]. Excess release of glutamate was found in schizophrenia patients [77] and especially in schizophrenic patients with cognitive impairment and negative symptoms [78]. Many genes modified in the brains of patients with schizophrenia are associated with glutathione and oxidative stress pathways [79]. The data provide evidence for epigenetic and intrinsic factors on oligodendrocyte dysfunction in schizophrenia.

Frontal and temporal lobe white matter volumes continue to increase in humans throughout the first five decades of life [80–82]. The convergence of natural and genetic risk factors on this area in schizophrenia and bipolar disorder may help to explain the apparent vulnerability of oligodendrocytes in these disorders [69]. Recent studies suggest that abnormal development of oligodendrocytes is involved in the pathophysiology of schizophrenia. Katsel et al. [83] suggest that oligodendroglial deficit in schizophrenia might be associated with disruption of normal patterns of cell cycle gene and protein expression. Barley et al. [84] found that genes expressed after the terminal differentiation of oligodendrocytes tended to have lower levels of mRNA expression in subjects with schizophrenia compared to normal controls. Kern et al. [85] reported deficits in the expression of oligodendrocyte and myelin genes associated with a reduction in the number of oligodendrocytes and with abnormalities of cell cycle markers in some brain regions in schizophrenia.

Some of studies indicate that chronic treatment with antipsychotics may affect brain morphology [86–88]. However, we found no effect of neuroleptic treatment on Nv of oligodendrocytes in schizophrenia and bipolar disorder groups and revealed a lack of age-related increase in this parameter in major depression group without neuroleptic treatment. On the other hand, protective effects of neuroleptics on oligodendrocytes have been reported [89, 90]. Thus, we suggest that reported reductions in oligodendrocyte density in schizophrenia and mood disorder are related to the disease.

Oligodendrocytes form after wave of neurogenesis, and they show a selective vulnerability to cell death stimuli depending on their stage of development [91]. Gritti et al. [92] found age- and region-dependent quantitative changes in the cell composition of neural stem cells progeny (decreased quantity of neurons and oligodendrocytes; increased amount of astroglial cells). Aging had an effect on the morphological feature, number, and developmental regulation of oligodendroglial progenitors in rat CNS [93]. It is of interest to note that neural stem cell proliferation is decreased in schizophrenia, but not in depression [94], and haloperidol stimulate proliferation of oligodendrocytes precursors [90]. Benes [95] suggested delayed myelination in the prefrontal cortex in patients with schizophrenia. Akbarian et al. [96, 97] reported a maldistribution of the interstitial neurons in both prefrontal and temporal white matter in schizophrenia. The data suggested that neurodevelopmental abnormalities were present in at least a subgroup of psychiatric patients. Since young schizophrenia patients in the present study demonstrated decrease in Nv of oligodendrocytes, though no significant, we suppose that the deficit of oligodendrocytes might start during brain development and continue to become significant in elderly patients with schizophrenia.

Several limitations of the study must be noted. First, mean age in bipolar disorder was less than in schizophrenia group. Second, samples need to be comprised of both males and females, as there is clear evidence to suggest that the timing, course, and clinical features of schizophrenia are manifested quite differently in males and females, and this may be related to brain differences. Future studies will also need to include an evaluation of first episode psychotic patients, so as to provide a sample of patients who have limited exposure to psychotropic medications. It will also be important to evaluate a representative sample of normal controls across age, as white matter changes with age, and such changes may be quite different in pathological populations such as schizophrenia. Future DTI studies need also to include other imaging techniques, which highlight white matter pathologies, such as proton spectroscopy, magnetization transfer techniques, and relaxation times measurements. Moreover, such studies should be conducted in concert with functional MRI and PET imaging in order to characterize and to understand more fully both functional and structural abnormalities in schizophrenia. Although gender ratios were not precisely matched, previous studies have failed to correlate gender and measures of diffusion tensor imaging.

## 4. Conclusion

In conclusion, the study highlights the importance of inclusion of age effects in imaging and postmortem studies for further studies of white matter abnormalities in major psychiatric disorders.

##  Conflict of Interests

The authors have no conflict of interest with any commercial or other associations in connection with the submitted article.

## Figures and Tables

**Table 1:** Demographic data of SFNC brain collection.

	Schizophrenia	Bipolar disorder	Major depression	Normal controls
	*n* = 15	*n* = 15	*n* = 15	*n* = 15
Age (mean ± SD)	44.5 ± 13.1	42.3 ± 11.7	46.5 ± 9.3	48.1 ± 10.7
Gender	9M, 6F	9M, 6F	9M, 6F	9M, 6F
Duration of disease (years) (mean ± SD)	20.6 ± 11.6	20.9 ± 10.2	12.7 ± 11.1	0
Age of onset (mean ± D)	23.2 ± 7.9	21.5 ± 8.3	33.9 ± 13.3	
PMI (h) (mean ± SD)	33.7 ± 14.6	32.5 ± 16.1	27.5 ± 10.6	23.7 ± 9.9
Side of brain	6R, 9L	8R, 7L	6R, 9L	7R, 8L
TF (m) (mean ± SD)	11.2 ± 8.4	9.6 ± 3.6	8.4 ± 6.6	4.4 ± 3.8

M: male; F: female; W: white; B: Black; A: Asian; PMI: postmortem interval in hours; L: left; R: right; TF: time in formalin in months.

**Table 2:** Demographic data of MHRC brain collection.

	Controls (*n* = 32)	Schizophrenia (*n* = 32)
Age (mean ± SD)	55.9 ± 15.2	51.4 ± 13.9
Gender	16M, 16F	19M, 13F
Duration of disease (years) (mean ± SD)	—	24.9 ± 10.5
Age of onset (mean ± SD)	—	26.7 ± 10.6
PMI (h) (mean ± SD)	6.0 ± 0.9	10.1 ± 7.7
TF (m) (mean ± SD)	1.3 ± 0.3	1.3 ± 0.4

M: male; F: female; PMI: postmortem interval in hours; TF: time in formalin in months.

**Table 3:** Regression of Nv of oligodendrocytes on age in BA9.

	*F*	*df*	*P*
Controls			
layer VI	10.05	1.13	.007
white matter	5.48	1.13	.03
Schizophrenia			
layer VI	2.15	1.13	.16
white matter	0.14	1.13	.7
Bipolar disorder			
layer VI	0.51	1.13	.5
white matter	4.63	1.13	.051
Major depression			
layer VI	0.01	1.13	.9
white matter	1.34	1.13	.26

**Table 4:** Regression of Nv of oligodendrocytes on age in BA10.

	*F*	*df*	*P*
Controls			
layer VI	6.55	1.30	.015
white matter	20.44	1.30	<.001
Schizophrenia			
layer VI	0.02	1.30	.9
white matter	0.90	1.30	.34
